# Using measures of quality of care to assess equity in health care funding for primary care: analysis of Indonesian household data

**DOI:** 10.1186/s12913-022-08739-z

**Published:** 2022-11-14

**Authors:** Manon Haemmerli, Augustine Asante, Dwidjo Susilo, Aryana Satrya, Rifqi Abdul Fattah, Qinglu Cheng, Soewarta Kosen, Danty Novitasari, Gemala Chairunnisa Puteri, Eviati Adawiyah, Andrew Hayen, Lucy Gilson, Anne Mills, Viroj Tangcharoensathien, Stephen Jan, Hasbullah Thabrany, Virginia Wiseman

**Affiliations:** 1 Department of Global Health and Development, London School of Hygiene & Tropical Medicine, London, UK; 2 School of Population Health, University of New South Wales, Sydney, Australia; 3 Faculty of Public Health, University of Indonesia, Jakarta, Indonesia; 4 Department of Management, Faculty of Economics, University of Indonesia, Depok, Indonesia; 5 Centre for Social Security Studies, University of Indonesia, Jakarta, Indonesia; 6 Kirby Institute, University of New South Wales, Sydney, Australia; 7 Independent Consultant, Jakarta, Indonesia; 8 Centre for Health Economics and Policy Studies, University of Indonesia, Jakarta, Indonesia; 9 School of Public Health, University of Technology Sydney, Sydney, Australia; 10 Health Policy and Systems Division, School of Public Health, University of Cape Town, Cape Town, South Africa; 11 International Health Policy Programme, Ministry of Public Health, Nonthaburi, Thailand; 12 The George Institute for Global Health, University of New South Wales, Sydney, Australia; 13 Faculty of Medicine and Health, School of Public Health, The University of Sydney, Sydney, Australia; 14 ThinkWell Indonesia, Jakarta, Indonesia

**Keywords:** Quality of care, Health financing, Benefit-incidence analysis, Indonesia

## Abstract

**Background:**

Many countries implementing pro-poor reforms to expand subsidized health care, especially for the poor, recognize that high-quality healthcare, and not just access alone, is necessary to meet the Sustainable Development Goals. As the poor are more likely to use low quality health services, measures to improve access to health care need to emphasise quality as the cornerstone to achieving equity goals. Current methods to evaluate health systems financing equity fail to take into account measures of quality. This paper aims to provide a worked example of how to adapt a popular quantitative approach, Benefit Incidence Analysis (BIA), to incorporate a quality weighting into the computation of public subsidies for health care.

**Methods:**

We used a dataset consisting of a sample of households surveyed in 10 provinces of Indonesia in early-2018. In parallel, a survey of public health facilities was conducted in the same geographical areas, and information about health facility infrastructure and basic equipment was collected. In each facility, an index of service readiness was computed as a measure of quality. Individuals who reported visiting a primary health care facility in the month before the interview were matched to their chosen facility. Standard BIA and an extended BIA that adjusts for service quality were conducted.

**Results:**

Quality scores were relatively high across all facilities, with an average of 82%. Scores for basic equipment were highest, with an average score of 99% compared to essential medicines with an average score of 60%. Our findings from the quality-weighted BIA show that the distribution of subsidies for public primary health care facilities became less ‘pro-poor’ while private clinics became more ‘pro-rich’ after accounting for quality of care. Overall the distribution of subsidies became significantly pro-rich (CI = 0.037).

**Conclusions:**

Routine collection of quality indicators that can be linked to individuals is needed to enable a comprehensive understanding of individuals’ pathways of care. From a policy perspective, accounting for quality of care in health financing assessment is crucial in a context where quality of care is a nationwide issue. In such a context, any health financing performance assessment is likely to be biased if quality is not accounted for.

## Background

Almost without exception, health systems worldwide provide health services that vary in terms of quality and access in ways that invariably favour higher income groups [1]. This occurs even in countries which have ostensibly achieved universal health coverage (UHC) and against a background of longstanding recognition of this type of disparity, countries are urged to ‘aim for affordable UHC and access for all citizens on the basis of equity and solidarity’ [2]. Nonetheless many low- and middle-income countries (LMICs) are implementing reforms to promote equity in health financing and delivery as a pathway towards UHC [3]. Measuring the distributional impact of these reforms is a high priority for these countries [4].

‘Benefit Incidence Analysis’, or BIA, is the traditional approach to estimating the distributional impact of government spending on health care [5]. It uses information on costs and the utilisation of health services to estimate the distribution of public spending across different socio-economic groups. BIA seeks to answer the question: who benefits from public expenditures on health care and by how much? Put differently, BIA measures by how much the income of a household would have to be raised if the household had to pay for the subsidized health services at full cost [6]. In practice, BIA studies estimate “benefits” or “public subsidies” to service users, who are typically ranked by socioeconomic status or some other variable of interest including geographical area, ethnic group or gender [7]. While BIA has traditionally focused on distribution of public sector subsidies, the analysis is increasing being extended to the private sector because of the growing and important role of the private health sector.

A key strength of a BIA is that it can provide a simple and transparent approach to assessing the extent to which public health spending benefits the poor. The approach, however, is not without its limitations. A key one that has been flagged by analysts is the failure to take account of variations in the quality of health services received by different individuals, leading to a potential under/over-estimation of the subsidy [8]. Increased evidence that the poorest segments of the population receive poorer quality of care [9], means that failing to take account of the quality of care in BIA could lead to a biased picture of who benefits most from government health spending.

Recently, Asante et al. attempted to address this critical methodological gap by introducing a quality score into the computation of BIA [8]. They developed a proxy measure for quality using area level deprivation indicators (availability of water, electricity, energy source for cooking, education, etc.). One limitation of their approach (acknowledged by the authors) is the use of deprivation indicators that are not directly related to health care quality. Second, they used area-level indicators by averaging the quality measures at the district level; this could not only mask variations in the quality of health services at the sub-district level, but most importantly across facilities used by individuals with varying socio-economic status (SES). In this paper, we address this important issue using data from linked household and primary health care facility surveys in Indonesia. We aim to provide a worked example of how to apply the quality-weighted BIA methodology using facility-level quality measures linked to individual utilization data.

## Methods

### Study setting

In 2014, Indonesia took a significant step towards UHC by implementing a comprehensive national social health insurance scheme, known as the Jaminan Kesehatan Nasional (JKN), to make health care available to its entire population [10]. The JKN brings together all major existing health insurance schemes under a single agency - the Social Security Agency for Health (BPJS-Health) - and was made mandatory for all Indonesians. Since the introduction of the JKN, Indonesia has made significant progress, moving from 46.5% of the population covered in 2014 to 83% as of May 2019 [11]. This makes the Indonesian Social Health Insurance (SHI) scheme one of the biggest single payer system in the world. Under the JKN, members must register with a contracted public and private primary care provider. The BPJS-Health pays these providers by capitation for outpatient services [11], and the capitation amount differs based on the total number of practitioners, the ratio of practitioners to beneficiaries, and operating hours.

### Utilisation data

We used data from a cross-sectional household survey (ENHANCE Survey) of 7500 households conducted in 10 provinces of Indonesia at the beginning of 2018. Details of the sampling methodology have been published elsewhere (https://equityhealthj.biomedcentral.com). Individuals were asked about their health seeking behaviour, including the name of the private or public outpatient facilities they have visited in the previous month, their socio-economic background, as well as their health insurance status. Those who reported being enrolled in health insurance could fall into either of these categories: individuals considered poor whose insurance contribution is fully subsidised (JKN-PBI group), individuals who need to contribute either via their payroll or to self-enrol and pay premium contributions (JKN non-PBI group) and those enrolled in insurance schemes administered by the local government (Jamkesda group).

### Health facility data

The sampling frame for the health facility survey was drawn from information provided by households in the ENHANCE Survey on the name of the primary health care facilities they visited in the previous month. Due to limited time and budget constraints, we could not collect information from all the facilities mentioned in the ENHANCE Survey. Instead, in each sub-district, we selected up to three facilities that were most frequently mentioned by respondents. All the facilities selected were under contract with BPJS-Health and receiving public subsidies (in the form of capitation payments) to provide services to JKN patients. These were either public health centres (Puskesmas), or private clinics. In each facility, the person in-charge was interviewed about general characteristics, infrastructure, and availability of supplies, equipment and drugs.

### Cost data

To estimate the unit cost of health services, we used the JKN claims data for 2018 obtained from BPJS-Health and data on capitation payments made to primary health care facilities for the same year. We estimated that the unit cost of one visit in a public health centre was Rp 40,000 (~US$2.8), while a visit in a private clinic was Rp 60,000 (~US$4.2). Out-of-pocket (OOP) payments were derived from the household survey, where individuals were asked about the amount they spent during their last outpatient visit.

### Measures of socio-economic status

We developed a standard asset-based measure of socio-economic status, using data on the ownership of a range of durable assets (e.g. car, refrigerator and television), housing characteristics (e.g. material of dwelling floor, roof and walls and main cooking fuel) and access to basic services (e.g. electricity supply, source of drinking water and sanitation facilities) [12]. While income and consumption are the most direct measures of socio-economic status, we used an asset-index in this study for its ease of data collection, ease of computation and applicability to the Indonesian context, where the size of the informal sector is high. Principal component analysis (PCA) was used to estimate wealth levels using the asset indicators [12, 13]. The basic idea of PCA is to replace a set of correlated variables with a set of uncorrelated “principal components” which represent unobserved characteristics of the population. The principal components are linear combinations of the original variables and the weights are derived from the correlation or covariance matrix (depending on whether the data have been standardised) [12]. It is assumed that the first principal component, which explains the most variance among the data, represents household wealth.

### Measure of health care quality

Donabedian’s framework for assessing quality of care describes healthcare service delivery as a continuum that includes structure, process and outcomes [14]. According to Donabedian, *structural quality* consists of human and key material resources such as infrastructure, equipment, drugs, medical supplies, communication, and transport. *Process quality* assesses whether what is known to be “good” medical care has been applied. Evidence-based care includes systematic patient assessment, accurate diagnosis, provision of appropriate treatment and technical competence in the provisions of diagnostic and therapeutic procedures, continuity of care, and appropriate patient counselling. *Health outcomes* refer to the ultimate improvement of health in terms of recovery, restoration of functions and survival.

In this study, we measured structural quality through the use of a supply-side readiness (SSR) index. The indicators of SSR were derived from the Service Availability and Readiness Assessment (SARA) tool [15]. Among the many indicators collected as part of the SARA survey, the “*general service readiness”* section collects information on the potential of health facilities to provide basic health care interventions. Following the SARA methodology, indicators were classified into five general service readiness domains (basic amenities, basic equipment, infection prevention, essential medicines and diagnostic capacity) (Table 1) and coded as binary variables, 1 indicating the presence of the indicator as reported by the provider, and 0 indicating non-availability. Each domain was associated with a score based on the percentage of items available. For each facility, an overall SSR score comprised between 0 and 1 was calculated based on the mean score across the five domains.

**Table 1:** Indicators for general service readiness used in analysis

*Domains*	*Indicators*
**Basic amenities (8)**	Physical access, toilet facilities, examination room with air conditioning, waiting room, internet connection, computer, running water, emergency room
**Infection prevention (4)**	Safe storage and disposal of infectious waste, safe storage and disposal of sharps, latex gloves, single use syringes.
**Basic equipment (5)**	Blood pressure meter, thermometer, baby scale, adult scale, and stethoscope.
**Essential medicines (21)**	Amlodipine tablet or alternative calcium channel blocker, Amoxicillin, Ampicillin, Aspirin, Beta blocker, Beclometasone inhaler, Carbamazepine, Enalapril tablet or alternative ACE inhibitor, Fluoxetine, Gentamicin injection, Glibenclamide tablet, Haloperidol, Insulin regular injection, Magnesium sulphate injectable, Metformin, Omeprazole or alternative, Oral rehydration solution, Salbutamol inhaler, Simvastatin or other statin, Thiazide, Zinc sulphate.
**Diagnostic capacity (8)**	Malaria rapid test, syphilis rapid test, HIV rapid test, pregnancy test, haemoglobin and blood count, blood glucose estimation, urine glucose test strips, urine protein test strips.

### Quality-weighted BIA

In this analysis, we restricted our sample to the individuals who could be linked to their health facility of choice, in which facility data was collected. The various steps and data required to conduct a BIA have been described in detail elsewhere [16, 17]. In traditional BIA, the unit subsidy received by each individual is represented by the unit cost incurred by the provider in delivering the service minus any fees paid by the user to the provider in using the service, that is:

1 $${S}_i=\left({C}_i-{F}_i\right)=\left({c}_i{q}_i-{f}_i{q}_i\right)={q}_i\left({c}_i-{f}_i\right)={q}_i{s}_i$$

Where *S*_*i*_ is the subsidy captured by individual i at the facility visited, *C*_*i*_ is the unit cost incurred by the provider at the facility in providing the services to individual *i*, *F*_*i*_ is the total fee paid by individual *i* to the provider, *q*_*i*_ is the quantity of services consumed within a month and *c*_*i*_, *f*_*i*_, *s*_*i*_ are the unit cost, fee and subsidy, respectively [18]. In cases where the computation of individual subsidies yielded negative figures, the values were set to zero. As unit costs vary between public and private facilities, individual subsidies must first be computed separately, and then the total subsidy computed as the sum of the subsidies for public and private health visits. Total subsidies were annualised by multiplying the monthly figures by 12. We first ran standard BIA using unadjusted subsidies by ranking households according to their level of wealth, and by estimating the distribution of the subsidies across income groups. Concentration curves (CCs) and concentration indices (CIs) were used to summarise the degree of inequality in the distribution of public health subsidies. Sampling weights have been applied to reflect the probability of each individual being sampled within each province, district, sub-district and village.

According to the Asante et al. framework, the quality-adjusted subsidies *WS*_*i*_ can be expressed in the following way:

2 $${WS}_i={S}_i{x}_i$$

Where *x*_*i*_ is the SSR score of the facility that individual *i* visited, and *S*_*i*_ is the unadjusted subsidy from (1). The quality-weighted BIA was run using the quality-adjusted subsidies as above, and results were compared with the standard BIA approach.

### Comparison with level of need

In traditional BIA, the distribution of public subsidies for health services is usually compared with the distribution of the need for health care in order to have a more complete picture of the degree of equity in the system [17]. Several national surveys in LMICs include questions on self-assessed health (SAH) that can be used to proxy health care need [19]. We therefore used a similar approach. In the ENHANCE survey, individuals were asked to rate their general health status. A four-point scale was developed with the following response options: “very good”, “good”, “bad” and “very bad”. Anyone who rated his/her health as ‘bad’ or ‘very bad’ was considered to be in need of health care. The distribution of unadjusted and quality-adjusted subsidies was then compared with the distribution of the need for health care, using SAH as a proxy for need.

## Results

Table 2 describes the basic characteristics of individuals in our sample. In total, we managed to link 784 individuals with 51 health facilities they visited, which represents about 19% of all the individuals in the sample who reported seeking primary health care in the previous month. Table 3 describes the health facilities surveyed: 84% were public health centres, and 16% private clinics. 37% offered inpatient services, and about half were open 24 hours a day. The average catchment of a health facility was 35,000 persons. All facilities were contracted with the BPJS-Health and therefore provided subsidised services to JKN patients. Quality scores were relatively high across all facilities, with an average of 82%. Scores for basic equipment were highest, with an average score of 99% (range 60 to 100%) compared to essential medicines with an average score of 60% (range 20 to 85%).

**Table 2:** Characteristics of individuals

Variable	Mean	SD	min	max
***Individual characteristics***	*N* = 645			
Area of residence is urban	68.6%			
Age (years)	30	24	1	96
Gender is female	59.2%			
Wealth quintile				
1	24.8%			
2	25%			
3	18.1%			
4	16.4%			
5	15.7%			
Number of people in the household	4.8	1.8	1	12
Insurance ownership				
JKN (PBI)	39%			
JKN (non-PBI)	23%			
Jamkesda	7%			
Private	1%			
No insurance	29%			
***Health seeking behaviour***				
Distance to health facility (as reported in km)	2.0	2.1	0.01	15
Time to reach health facility (as reported in min)	11.6	6.9	1	60
Out-of-pocket payments (as reported in IDR)	12,852	68,863	0	1,000,000

^a^*IDR* Indonesian rupiah. 1$ ~ 14,000 IDR in 2018, *PBI* insurance for the poor

**Table 3:** Characteristics of the health facilities

Variable	N	Mean	SD	min	max
***Health facilities characteristics***	*N* = 51				
Sector of care is public	43	84%			
Inpatient facility available	19	37%			
Catchment area	51	35,874	22,155	1995	103,904
Open 24 h on weekdays	24	47%			
Accreditation status					
No accreditation	15	27.4%			
Basic	11	21.6%			
Intermediate	15	29.4%			
Advanced	8	15.7%			
Full	2	3.9%			
Contract with BPJS	51	1			
***Quality scores***					
Basic amenities	51	77%	0.15	0.37	1
Infection prevention	51	98%	0.08	0.5	1
Basic equipment	51	99%	0.06	0.6	1
Essential medicines	51	60%	0.15	0.2	0.85
Diagnostic capacity	51	76%	0.26	0	1
Overall readiness score	51	82%	0.09	0.43	0.92

Table 4 presents the distribution of unadjusted and quality-adjusted subsidies. For the unadjusted subsidies, we found that the distribution of subsidies in the public sector was pro-poor (CI = − 0.04), while the distribution of subsidies in the private sector was significantly pro-rich (CI = 0.37). The overall distribution of subsidies was slightly pro-rich, but the CI was not statistically significant (0.032). When adjusting for quality, we found that the distribution of subsidies in the public sector became slightly less pro-poor (CI = − 0.03), while the distribution of subsidies in the private sector became more pro-rich (CI = 0.48). Overall the pro-rich distribution of total subsidies became statistically significant (CI = 0.037).

**Table 4:** Distribution of unadjusted (top) and quality-adjusted subsidies (bottom) across wealth groups

		***Unadjusted subsidies by wealth group***					
Wealth group	Mean quality score	Total amount of subsidy in public sector	% total subsidy in public sector	Total amount of subsidy in private sector	% total subsidy in private sector	Total unadjusted subsidies	% of total subsidies
Q1	0.81	110,000,000	25.1	4,320,000	6.9	114,320,000	22.9
Q2	0.82	117,000,000	26.9	10,100,000	16.3	127,100,000	25.6
Q3	0.82	77,600,000	17.8	5,760,000	9.3	83,460,000	16.7
Q4	0.83	74,800,000	17.1	11,500,000	18.6	86,300,000	17.3
Q5	0.84	54,200,000	12.4	30,200,000	48.8	84,400,000	17.0
**Concentration index**			-0.04 (0.02)*		0.37 (0.10)***		0.032 (0.02)
		***Quality- adjusted subsidies by wealth group***					
Wealth group	Mean quality score	Total amount of subsidy in public sector	% total subsidy in public sector	Total amount of subsidy in private sector	% total subsidy in private sector	Total quality-adjusted subsidies	% of total subsidies
Q1	0.81	90,200,000	24.9	2,900,000	7.6	93,100,000	23.3
Q2	0.82	97,000,000	26.8	3,970,000	10.5	101,000,000	25.3
Q3	0.82	63,900,000	17,6	3,348,000	8.8	67,200,000	16.8
Q4	0.83	62,400,000	17.2	3,966,000	10.4	66,400,000	16.6
Q5	0.84	46,400,000	12.8	23,800,000	62.7	70,200,000	17.5
**Concentration index**			-0.03 (0.02)		0.48 (0.13)***		0.037 (0.02)*

^*^p < 0.05

^**^p < 0.01

^***^p < 0.005

**Fig. 1:**
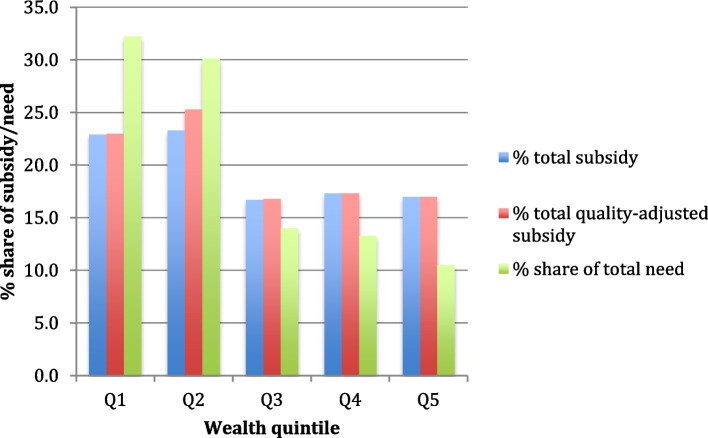
Overall public adjusted- and unadjusted total subsidies and level of health need

Figure 1 compares the mean level of need with the distribution of subsidies across wealth quintiles. The level of need was highest among the poorest quintiles, and the distribution of public subsidies was not proportional to the distribution of the need for health care. In other words, the poorest groups in our sample did not receive their fair share of public subsidies when considering their level of need. The level of inequality was slightly higher when using quality-adjusted subsidies, but the magnitude was small.

## Discussion

This study provides a worked example of how to apply a quality-weighted BIA methodology, and we summarise the main steps in Table 5 below. Our findings from Indonesia show that the distribution of subsidies for public primary health care facilities became less ‘pro-poor’ and subsidies for private primary health care facilities became more pro-rich after accounting for quality of care. The magnitude or the difference between the distributions of quality-adjusted and unadjusted subsidies was not large, and we believe that the gap between the two distributions is likely to be underestimated. As our measures of quality remain limited to structural indicators and do not include process or outcomes measures, we did not find large variations in quality across study sites though availability of essential medicines has the lowest score. More sensitive measures of quality and/or the inclusion of higher-level facilities such as tertiary care hospitals would have provided a more realistic picture of quality-adjusted subsidies in Indonesia, and potentially greater differences in quality-adjusted and unadjusted subsidies. Additionally, recent efforts to increase quality of care in Indonesia, such as accreditation of primary health care facilities, have certainly led to a standardisation of the basic level of infrastructure and equipment However, this study is aimed at illustrating in practice how to apply quality weights in BIA studies rather than producing precise quantitative estimates.

**Table 5:** Step-by-step procedure to run a quality-weighted BIA: adapted from [17]

*Step*	*Description*
**1: Preparing household data**	Select a measure of living standard or socio-economic status (SES) and rank the population from poorest to richest; Estimate the utilization of different types of health service by individuals/different socio-economic groups (services such as primary level clinics, district hospitals, regional hospitals and central hospitals in the case of public sector services; if considering private sector services as well, categories such as general practitioners, specialists, retail pharmacies and private hospitals); Register and list the names of the health facilities individuals visited, and use this list as a sampling frame for the facility survey.
**2: Preparing facility data**	Quality indicators should be as detailed as possible and should include structural, process and outcome measures of quality. Observed quality indicators are preferred over self-reported indicators. Develop a quality score: quality indicators should be aggregated into a single measure. Different weighting schemes are possible, although equal weights are easier to interpret.
**3: Linking both datasets**	Household and facility data should be linked by using a unique facility identifier number
**4: Estimate quality-adjusted subsidies**	Unadjusted subsidies are computed as in traditional BIA. For each individual, estimate the quality-adjusted subsidy by multiplying the unit subsidy by the quality score of the facility visited.
**5: Assess equity in distribution of health subsidies**	Aggregate the distribution of both unadjusted and quality-adjusted subsidies expressed in monetary terms, across different types of health service for each individual/socio-economic group.
**6: Comparison with level of need**	Compare the distributions of both unadjusted and quality-adjusted subsidies to some target distribution (e.g. relative to need for health care).

Indonesia has been the focus of few BIA studies [19]. The first study, published over 30 years ago, showed that primary health care was mildly progressive but hospital care was disproportionately used by the better-off [20]. Similar results were reported in 2001 [21]. A comparative analysis of Asian countries found that in Indonesia, the richest 20% of the population received more than 30% of the total subsidies, and that the distribution of health care utilisation was more pro-poor than the subsidy distribution [22]. The fourth study examined the marginal effects of decentralized public health spending on the benefit incidence, when the authority to manage public spending for health and other sectors was devolved to the district level [23]. This study found that increased public spending at the district level improved the targeting of public funds to the poor by increasing their utilisation of services and also their share of public expenditure. However, the authors concluded that effort to increase the use of health services by the poor was necessary, and that demand-side interventions, such as price subsidies or social health insurance, were needed.

To our knowledge, none of these studies took into account the quality of care that patients received. More recently, Sambodo et al. measured the benefit incidence of healthcare funding under JKN, taking into account regional variation in unit costs across districts [24]. As both primary and secondary care providers are paid prospectively and proportionally to the intensity of their activity under the JKN system, better-equipped service providers are more likely to receive larger provider payments. Sambodo et al. found that the distribution of benefits favoured the wealthier groups, but most importantly that standard BIA using national unit costs underestimates regional disparities in healthcare funding, and therefore underestimates the inequality in the benefit distribution. If one assumes that the variation in unit costs reflects the variations in quality of care (especially availability of basic amenities, basic medical equipment, essential medicines and diagnostic tools), then our findings are consistent with theirs in the sense that the level of inequality in the benefit distribution is underestimated if quality is not accounted for. However, this assumption is unlikely to hold if higher provider payments are not correlated with higher quality, but instead are reflective of higher level of inefficiency; hence the need to account for quality using robust measures.

A major strength of our analysis lies in the fact that we were able to link individuals with the facilities they visited. In most studies, data linkage is not possible at the individual level, since conducting a facility survey alongside a household survey can be resource-intensive. Some limitations should also be acknowledged. Due to time and budget constraints, only 51 primary healthcare facilities could be surveyed, and therefore data on quality was collected in only a fraction of health facilities that individuals in our survey visited in the previous month, making the picture incomplete. However, this study represents a methodological advancement by introducing quality weights into the BIA framework and we hope future studies will be able to validate these results with larger datasets. Another limitation is the use of supply-side readiness scores as a proxy for quality which do not take into account e.g. health systems responsiveness and people’s expectations [25]. Careful interpretation is needed since the concept of quality of care is considerably broader and more complex than the measure used here [26]. Inputs such as infrastructure, equipment, medicines, and diagnostic tests, are just one element or prerequisite to the provision of good quality care [27]. Finally, while concerns have been raised about the use of subjective measures of health care need, they tend to be more readily available to researchers than objective measures and have been validated in the analyses of inequalities in health [28].

From a methodological perspective, one of the challenges of accounting for quality of care in BIA is the lack of adequate data from LMIC settings or standardized measurement of quality, since incomplete and unreliable quality data are common, and they often poorly capture process and outcome measures of care [29]. Researchers often rely on secondary datasets made available through global agencies such as the WHO, World Bank or United States Agency for International Development. The Indonesian Family Life Survey, for example, conducts health facility surveys that incorporate various indicators, including structural and process indicators, which can be used to assess quality of care. However, quality data is collected in only a fraction of health facilities that individuals visited in the previous month, making the picture incomplete. Routine collection of quality indicators that can be linked to individuals are needed to enable a more comprehensive understanding of individuals’ pathways of care, including the quality of services they receive.

The implications of this study go beyond the methodological aspect. From a policy perspective, accounting for quality of care in health financing assessment is crucial in a context where quality of care is a nationwide issue. Recently, the World Bank conducted an assessment of a nationally representative sample of 686 Indonesian public and private primary health care facilities. Their report highlights significant gaps in the readiness of primary health care facilities to deliver a basic level of quality of care [30]. Additionally, large geographical inequalities in the quality of care were detected. In such a context, any health financing performance assessment is likely to be biased if quality is not accounted for.

## Conclusion

Through this analysis, we have shown that accounting for quality in BIA studies may provide a more accurate picture of the level of inequality, since poor households may have no choice except to visit the lower quality health facilities in their communities. We recommend that future analysis looking at the level of inequality in the distribution of public health care subsidies should incorporate quality of care in order to get the most accurate picture of the health financing system. Table 5 provides ‘how to’ for future assessment of quality-adjusted BIA. Improvement of the method will lie in the scope of measurement (structural, process or outcomes) of quality of care using standardized indicators, as well as in the accuracy of linking individuals to the very facilities they reported visiting to avoid using area-level information. Policy should focus on strengthening and equalizing quality of care across all primary healthcare facilities, as recommended by the World Bank (World Bank Group, 2020).

## Data Availability

The datasets analysed during the current study are not publicly available since the data is still being analysed by the research team but are available from the corresponding author on reasonable request.

## Abbreviations

BIA: Benefit-incidence analysis
UHC: Universal Health Coverage
LMICs: Low-and middle-income countries
SES: Socio-economic status
JKN: Jaminan Kesehatan Nasional
SHI: Social Health Insurance
OOP: Out-of-pocket
PCA: Principal component analysis
SSR: Supply-side readiness
SARA: Service Availability and Readiness Assessment
CI: Concentration index
CC: Concentration curve
SAH: Self-assessed health
WHO: World Health Organisation
